# The Delivery of Multipotent Adult Progenitor Cells to Extended Criteria Human Donor Livers Using Normothermic Machine Perfusion

**DOI:** 10.3389/fimmu.2020.01226

**Published:** 2020-06-25

**Authors:** Richard W. Laing, Samantha Stubblefield, Lorraine Wallace, Valerie D. Roobrouck, Ricky H. Bhogal, Andrea Schlegel, Yuri L. Boteon, Gary M. Reynolds, Anthony E. Ting, Darius F. Mirza, Philip N. Newsome, Hynek Mergental, Simon C. Afford

**Affiliations:** ^1^NIHR Liver Biomedical Research Unit, Centre for Liver Research, College of Medical and Dental Sciences, Institute of Immunology and Immunotherapy, University of Birmingham, Birmingham, United Kingdom; ^2^Liver Unit, Queen Elizabeth Hospital, University Hospitals Birmingham NHS Foundation Trust, Birmingham, United Kingdom; ^3^Athersys Inc., Cleveland, OH, United States; ^4^ReGenesys BVBA, Leuven, Belgium

**Keywords:** liver transplantation, organ preservation, organ donation, mesenchymal progenitor cells, machine perfusion, stem cell therapy, immunomodulation, marginal donors

## Abstract

**Background:** Pre-clinical research with multi-potent adult progenitor cells (MAPC® cells, Multistem, Athersys Inc., Cleveland, Ohio) suggests their potential as an anti-inflammatory and immunomodulatory therapy in organ transplantation. Normothermic machine perfusion of the liver (NMP-L) has been proposed as a way of introducing therapeutic agents into the donor organ. Delivery of cellular therapy to human donor livers using this technique has not yet been described in the literature. The primary objectives of this study were to develop a technique for delivering cellular therapy to human donor livers using NMP-L and demonstrate engraftment.

**Methods:** Six discarded human livers were perfused for 6 h at 37°C using the Liver Assist (Organ Assist, Groningen). 50 × 106 CMPTX-labeled MAPC cells were infused directly into the right lobe via the hepatic artery (HA, *n* = 3) or portal vein (PV, *n* = 3) over 20 min at different time points during the perfusion. Perfusion parameters were recorded and central and peripheral biopsies were taken at multiple time-points from both lobes and subjected to standard histological stains and confocal microscopy. Perfusate was analyzed using a 35-plex multiplex assay and proteomic analysis.

**Results:** There was no detrimental effect on perfusion flow parameters on infusion of MAPC cells by either route. Three out of six livers met established criteria for organ viability. Confocal microscopy demonstrated engraftment of MAPC cells across vascular endothelium when perfused via the artery. 35-plex multiplex analysis of perfusate yielded 13 positive targets, 9 of which appeared to be related to the infusion of MAPC cells (including Interleukin's 1b, 4, 5, 6, 8, 10, MCP-1, GM-CSF, SDF-1a). Proteomic analysis revealed 295 unique proteins in the perfusate from time-points following the infusion of cellular therapy, many of which have strong links to MAPC cells and mesenchymal stem cells in the literature. Functional enrichment analysis demonstrated their immunomodulatory potential.

**Conclusion:** We have demonstrated that cells can be delivered directly to the target organ, prior to host immune cell population exposure and without compromising the perfusion. Transendothelial migration occurs following arterial infusion. MAPC cells appear to secrete a host of soluble factors that would have anti-inflammatory and immunomodulatory benefits in a human model of liver transplantation.

## Highlights

- Transplant surgeons are becoming more reliant on the use of marginal donor livers
- NMP-L provides the unique opportunity to deliver therapeutics to donor livers
- MAPC cells have beneficial immunomodulatory and anti-inflammatory effects
- Infusing MAPC cells via the hepatic artery results in consistent engraftment
- MAPC cells secrete a unique beneficial proteome that may improve outcomes.

## Introduction

The demand for donor livers overwhelms supply and in the UK, 19% of patients die or are removed from the list whilst waiting for a transplant (1). Strategies to improve the quality of high risk donor livers [531 rejected in the UK last year (1)] would increase the pool of transplantable livers and improve patient outcomes.

Multipotent adult progenitor cells (MAPC®) have been proposed as an immune-active treatment for a wide variety of conditions (2). They belong to the family of mesenchymal stem cells (MSC) but show a higher proliferative capacity and a broader differentiation potential (3). A distinct bone-marrow derived cellular population, they meet the formal criteria for designation as stromal stem cells in that they are plastic-adherent and express CD73, CD90, and CD105, in the absence of the hematopoietic markers CD14, CD34, CD45, and HLA-DR (4). They differ from MSC based on cellular phenotype (negative for CD140a, CD140b, alkaline phosphatase and express major histocompatibility complex class I at lower levels), size, transcriptional profile, and expansion capacity (5). Proof of concept of their efficacy has been demonstrated in animal models for the treatment of different conditions including graft versus host disease and in a porcine and human lung model of machine perfusion (6–11). Not only can they impair the induction of CD8+ cytotoxic T-lymphocyte function and supress T-lymphocyte proliferation (12), but MAPC cells and related mesenchymal stem cells (MSC) have been shown to reduce ischaemia reperfusion injury (IRI) and reduce the inflammatory response in solid organs (2, 10, 13, 14). These preclinical studies suggest that MAPC cells could exert their beneficial effects in a solid organ transplant model through immunomodulation by promoting immunological tolerance (9, 15–17).

Transplantation is the only curative option for patients with end-stage liver disease and the global shortage of suitable donor livers has been extensively reported (18, 19). The UK transplant activity data over the past decade (2008–2018) demonstrates a 54% increase in transplant activity (657 to 1014) (1). The increase in donor numbers over this period has been achieved through a 58% increase in livers donated following brain death (DBD) and a 257% increase in those donated following circulatory death (DCD) (20). Our own data shows a pre-transplant on-list mortality rate for priority patients of up to 40% (unpublished data). It is widely accepted that whilst the use of extended criteria DCD or marginal DBD liver grafts may provide additional organs for transplantation they are known to be associated with additional challenges (21–23). Given the significant clinical impact of these factors, there is an urgent clinical need to attempt to modulate the inflammatory and immune responses they induce.

Normothermic machine perfusion of the liver (NMP-L) is a novel technique whereby a donor liver graft is perfused at physiological temperature and pressure with a complex solution containing an oxygen carrier and other constituents (including colloid, electrolytes etc.) that aims to preserve the graft under physiological conditions *ex-situ*. It has been shown to be a superior to static cold storage as a method of organ preservation (24, 25), it also provides the unique opportunity to assess organ viability prior to transplantation (26–29). The potential use of NMP-L as a method of delivering cell-based and novel small molecule therapies aimed at improving the condition of extended criteria livers has been proposed (30) and is steadily gaining credence within the transplant community as experimental proof that concept data is emerging (31, 32). Despite examples in animal models, delivery of cellular therapy using machine has not been demonstrated in a human liver model (33–35).

The aims of this study were to (a) develop and demonstrate feasibility of NMP-L as a technique for delivering cellular therapy to extended criteria human donor livers; (b) determine the best vascular route for delivery and confirm the presence of cellular engraftment and (c) determine parameters that may reflect biologically functional activity imparted by the presence of the therapeutically administered MAPC cells.

## Materials and Methods

### Preparation of MAPC Cells

MAPC cells were provided by Athersys Inc. (Cleveland, Ohio, USA). The isolation and cultivation of these MAPC cells have been previously described (36). Cryovials containing ~10 × 10^6^ cells labeled with CellTracker™ Red CMTPX dye (Thermo Fisher Scientific Inc.) were thawed and prepared according to clinical protocols immediately prior to infusion into the donor liver (see supplementary information for protocols). Cellular concentrations and viability were determined using trypan blue dye exclusion and 50 × 10^6^ cells were made up to a final volume of 50 ml with 0.9% normal saline ready for infusion. Calculations of number of cells were based on clinical studies where cells were delivered systemically (150–600 million) and this was scaled down due to infusion into the target organ and in this case the right lobe of the liver (16).

### Source of Discarded Human Livers

The six donor livers included in this study were offered, accepted and retrieved with the initial intention to use them for clinical transplantation. They were procured by one of the UK's National Organ Retrieval Service teams using nationally agreed surgical protocols (National standards for organ retrieval from deceased donors (joint with NHSBT). Available from: http://www.bts.org.uk). Following assessment by either the retrieval or transplanting surgeon, the livers were declined by all UK transplant centers and consent-permitting, subsequently offered for research by the NHSBT co-ordinating office. Ethical approval for the study was granted by the National Research Ethics Service committee in London-Surrey Borders (reference number 13/LO/1928). Consent for the use of donor tissues for research was obtained by the specialist nurses in organ donation from the designated donor's next of kin.

### Preparation of the Donor Liver for NMP-L and MAPC Cell Infusion

**Figure 1:**
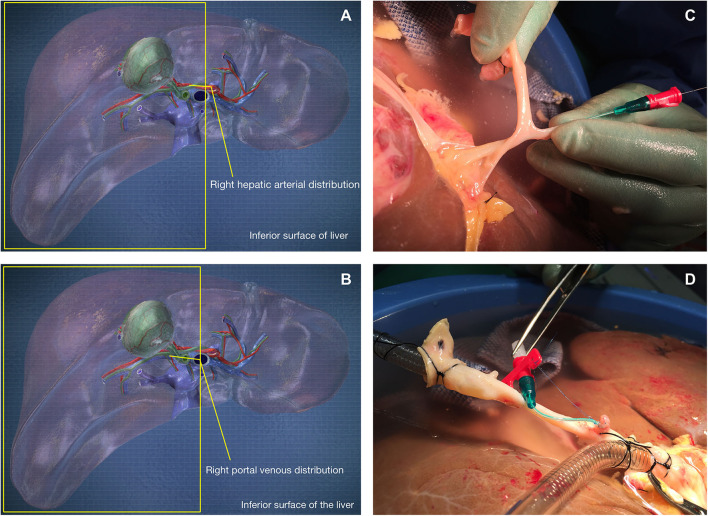
Cells were infused via the gastroduodenal arterial stump **(C,D)** into the right hepatic arterial branch **(A)** or directly into the right portal venous branch **(B)**.

On receipt of the donor liver, its preparation for NMP-L was initially analogous to clinical transplantation. A polyethylene Leadercath Arterial catheter [Vygon [UK] Ltd] was placed to permit infusion of cellular therapy into the right lobe either via the hepatic artery or portal vein. For arterial infusion the guidewire was passed through the gastroduodenal arterial stump and gently directed into the main branch of the right hepatic artery. For portal venous infusion the needle supplied was used to puncture the portal vein proximal to the bifurcation and the wire passed down the right portal venous branch. The catheter was then guided over the wire and into the appropriate vessel and secured using 5-0 prolene sutures. Cells were infused directly into the right lobe via either the right hepatic arterial branch or the right portal vein branch to create an internal control and gain information on engraftment of recirculating cells. A 3-way tap was attached to the catheter, flushed with 2 ml of Ringer's solution and set to the closed position. The distal end of the catheter was always placed in the main trunk of the right arterial or portal venous branch (Figure 1). Following insertion and securing of cannulae, The liver was placed into the machine reservoir and connected to a Liver Assist device (CE marked; Organ Assist, Groningen, The Netherlands) as previously described (29, 37).

### Infusion of MAPC Cells

MAPC cells were infused via syringe driver attached to the Vygon Leadercath catheter over 20 min into the right lobe via the hepatic artery (HA, *n* = 3; HA1, HA2, HA3 [1 DBD and 2 DCD]) or portal vein (PV, *n* = 3; PV1, PV2, PV3 [1 DBD and 2 DCD]) during the perfusion. The cells were infused as described initially after 4 h of perfusion (*n* = 2, first HA and PV infusion). Vascular flow characteristics were unaffected by the infusion, therefore subsequent infusions were performed after 1 h (*n* = 4, 2 HA, and PV infusions).

### Assessment of Physiology and Sample Collection Protocol

Flow rates, pressures, resistances and temperatures in the hepatic arterial and portal venous circuits were recorded every 30 min and specifically before, during and after cell infusions. Arterial and hepatic venous perfusion fluid was sampled every 30 min and immediately assessed using a Cobas b 221 point of care system (Roche Diagnostics, USA). Samples were also processed to permit the freezing of perfusate at −80°C. Livers that metabolized lactate to below 2.5 mmol/L within 2 h were termed “viable” as it is predicted that these livers have the metabolic capacity to function sufficiently following transplantation (28)—a hypothesis that was tested during the clinical pilot study as well as in the VITTAL trial (Viability Testing and Transplantation of Marginal Livers) which is now closed to recruitment (27, 38).

### Histological Assessment

Liver biopsies were taken from both the left and right lobes; on the back bench prior to the start of NMP-L, pre-cell infusion and at the end of the 6-h perfusion. Biopsies were fixed in formalin, embedded in paraffin and sections cut at 4 μm. The MAPC cells were identified by the CellTracker™ Red CMTPX dye and their biodistribution—related to their route of administration assessed using confocal microscopy. Three-color confocal microscopy (4',6-diamidino-2-phenylindole [DAPI] on the blue channel, CMTPX Red on the red channel and CD31 on the green channel (to identify vascular endothelium)) was used to demonstrate the presence and location of MAPC cells. The creation of virtual slides through imaging of whole tissue mounts was achieved using the ZEISS AxioScanZ.1 slide scanner and confocal microscopy was performed using the ZEISS LSM780 confocal microscope.

### Assessment of Soluble Markers in Perfusate Samples

#### Cytokine and Chemokine Analysis Using Multiplex Array

Perfusate samples from all perfusions at 4 time-points were analyzed using the 34-Plex Human ProcartaPlex™ Panel 1A multiplex kit (ThermoFisher Scientific Ltd.). The target list included Eotaxin/CCL11; GM-CSF; GRO alpha/CXCL1; IFN alpha; IFN gamma; IL-1 beta; IL-1 alpha; IL-1RA; IL-2; IL-4; IL-5; IL-6; IL-7; IL-8/CXCL8; IL-9; IL-10; IL-12 p70; IL-13; IL-15; IL-17A; IL-18; IL-21; IL-22; IL-23; IL-27; IL-31; Interferon gamma-induced protein 10 (IP-10/CXCL10); Monocyte chemoattractant protein-1(MCP-1/CCL2); Macrophage inflammatory protein-1 alpha (MIP-1 alpha/CCL3); MIP-1 beta/CCL4; RANTES/CCL5; Stromal cell-derived factor-1 (SDF1 alpha/CXCL12); TNF alpha; TNF beta/LTA. A “viable” liver that had not received MAPC cells and was transplanted as part of the clinical pilot study was used as a control. The multiplex assay was performed according to the manufacturers guidelines and run on a Luminex® 100™ System. Raw data were analyzed using Prism 8.0 for Mac OS X.

#### Proteomic Analysis of the Perfusate

Proteomic analysis of individual perfusate samples from four time-points was performed for each liver and compared to results from all other livers (*n* = 8) previously perfused with standard perfusate that had not received cellular therapy. This was to maximize the probability of identifying unique proteins in the MAPC cell perfused livers. Hemoglobin depletion of haemolysed samples using Hemoglobind (BioTech Support Group LLC, Monmouth Junction, NJ) was followed by trypsin-based liquid digestion, peptide cleaning, gradient separation and elution into a Linear Trap Quadropole (LTQ) Orbitrap Elite mass spectrometer for liquid chromatography (LC-MS/MS). Scan results were searched against Uniprot database. Protein-protein interactions (PPI's) and functional enrichments (FE's) were determined using the String database 2017 (https://string-db.org, String Consortium 2020) and Cytoscape (Cystoscape Consortium (39–41).

## Results

### Donor Demographics and Perfusion Parameters

**Figure 2:**
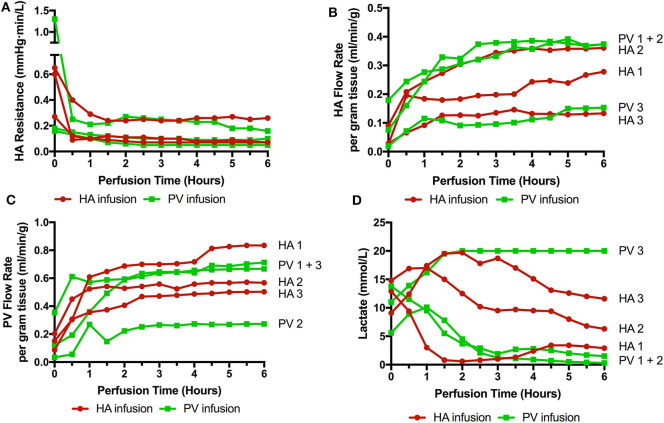
Perfusion parameters during 6 perfusions (HA1-3 cells infused via right hepatic artery. PV1-3 cells infused via right portal venous branch). **(A)** HA resistance; **(B)** HA flow rate adjusted for liver weight; **(C)** PV flow rate; **(D)** Lactate level over the course of the perfusion. 3 livers met viability criteria according to our Birmingham Machine Perfusion Group Viability Criteria. Two of the non-viable livers HA3 and PV3 also have very low arterial flow rates due to high intrinsic arterial resistances.

Six livers were perfused (2 DBD and 4 DCD) with a median donor age of 52.5 (35–71), cold ischemic time of 500 min (453–754), and donor risk index of 2.41 (1.58–3.22). Three received cells via the right hepatic artery and 3 via the right portal vein (1 DBD and 2 DCD in each group). The timing of infusions varied also. HA1 and PV1 received cells toward the end of the perfusion (infusions started at 4 h 40 mins and ran over 20 min, cells delivered with 1 h perfusion remaining) and in the remaining four livers (HA2, 3, and PV2, 3) the cells were infused after 40 min of perfusion and were delivered fully with 5 h of perfusion remaining. There were no significant detrimental effects on the perfusion parameters during cellular infusion and neither resistances or flow rates were adversely affected. Of interest, flow rates in the artery transiently increased by ~30% during all 3 arterial infusions but flows returned to normal shortly after stopping the infusions (data not shown). Arterial resistance and flow, portal flow and lactate can be seen in Figure 2.

### Histology and Confocal Microscopy

**Figure 3:**
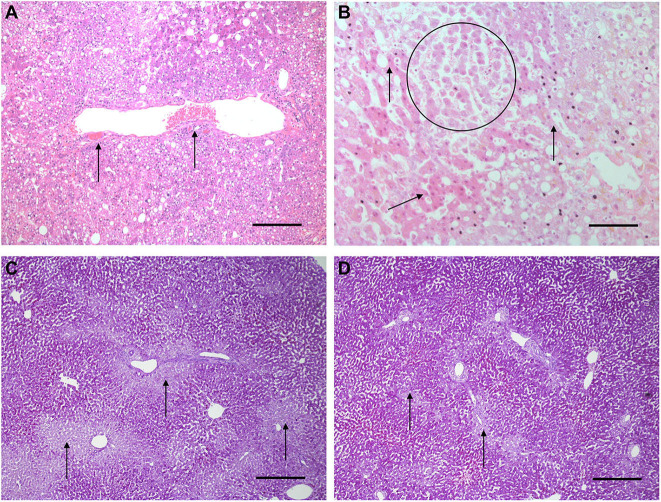
Light microscopy images of H&E **(A,B)** and periodic acid Schiff stains **(C,D)**. **(A,B)** are H&E stained sections from early NMP-Ls showing some histological abnormalities. Architecture of the liver parenchyma was well-maintained in those livers that were deemed viable. Liver PV3 **(B)** was severely steatotic and H&E stained sections demonstrated large droplet macrovescicular steatosis and loss of cohesion between hepatocytes in liver cell plates suggesting endothelial disruption. In those livers that met viability criteria, increases in glycogen storage were observed (Figures 3C,D). In HA3 (slide **A**), portal microvessels (arrows) are seen plugged by disintegrating red cells. Original objective × 10. Original objective × 10, scale = 100 μm. **(B)** 1 h after commencement of perfusion number PV3, loss of cohesion between hepatocytes in liver cell plates is observed (circle). Normal liver cell plates are arrowed. Original objective × 20, scale = 50 μm. **(C,D)** are periodic acid Schiff stained sections of liver PV1. **(C)** PV1 before NMP-L. **(D)** PV1 After 4 h of NMP-L. Glycogen stains as dark pink, arrows highlight pale glycogen depleted areas. It can be seen that there is less glycogen depleted pale areas after perfusion indicating that the hepatocytes have taken up glucose from the perfusate and metabolize it to glycogen. Original objective × 5 for both, scale = 200 μm.

**Figure 4:**
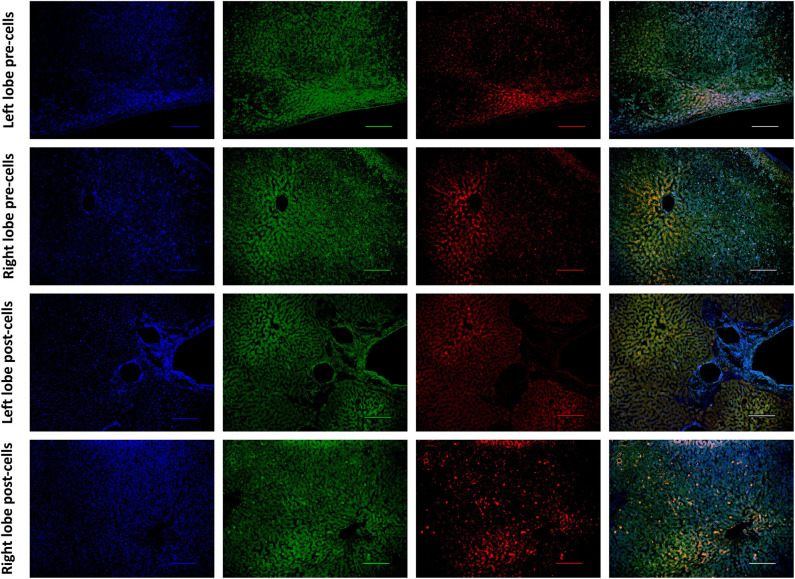
Confocal microscopy images showing representative tissue sections (as labeled) of livers infused with MAPC cells. Blue channel (Nucleic acid probe DAPI 345nm) (4',6-diamidino-2-phenylindole), Red channel (615 nm) CMTPX Red and green channel (FITC 495 nm). Four confocal microscopy panels (blue channel, green channel, red channel, and composite) of representative images of the left lobe **(A)** and right lobe **(B)** prior to MAPC cell administration and left lobe **(C)** and right lobe **(D)** 4 h after MAPC cell administration via the right hepatic artery. The cells are clearly seen in **(D)** fluorescing in the red and green channels and visible as orange cells in the composite image. Cells were never seen in any of the left lobe biopsies at 1 or 4 h after MAPC cell administration. A-D Objective × 10, scale =100 μm.

**Figure 5:**
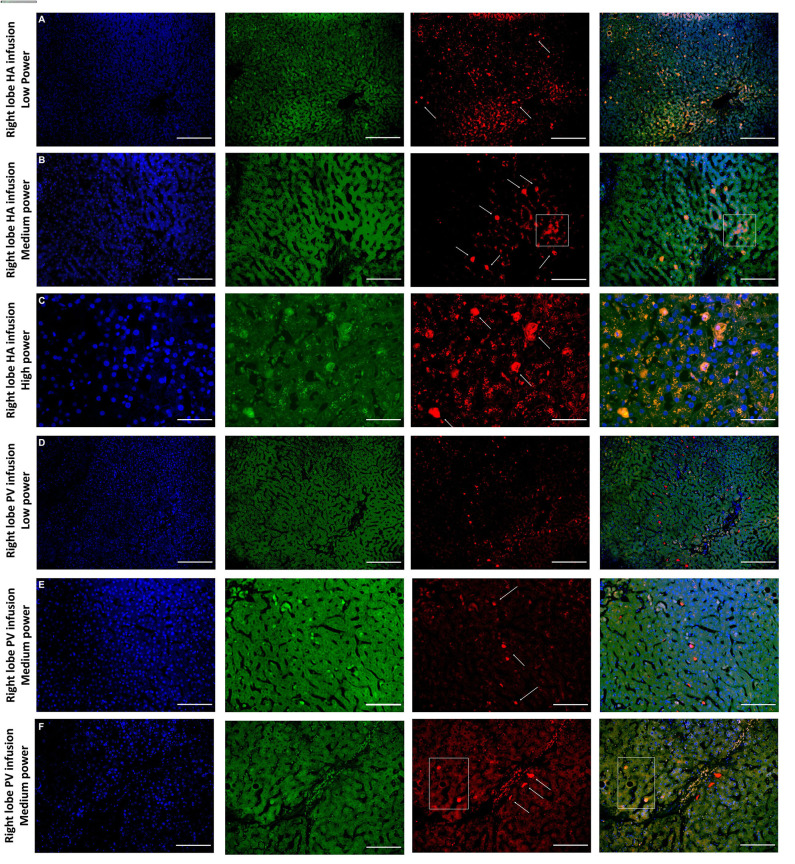
Confocal microscopy images showing representative tissue sections (as labeled) of livers infused with MAPC cells. Blue channel (Nucleic acid probe DAPI 345nm) (4',6-diamidino-2-phenylindole), Red channel (615 nm) CMTPX Red and green channel (FITC 495 nm). Six confocal microscopy **(A–F)** (blue channel, green channel, red channel, and composite) of representative images of the right lobe comparing route of delivery of the MAPC cells. **(A)** HA low power; **(B)** HA medium power; **(C)** HA high power; **(D)** PV low power; **(E)** PV medium power; **(F)** PV high power. **(A–C)** Demonstrate widespread delivery of MAPC cells which are visible (arrows) in both the green and red channel images suggesting a possible conformational change following engraftment which is more clearly demonstrated in Figures 6, 7. The square annotation in **(B)** shows the autofluorescence commonly seen in the red channel in liver tissue, however the granular pattern is clearly different to the solid appearance of the cells that fluoresce due to the CMTPX stain. **(C–E)** Demonstrate cells arrested within the sinusoids of the liver following administration via the right portal vein. These are much brighter in the red channel and they clearly reside within the vascular channels. In **(F)** there are two cells which appear similar to those in **(A–C)** suggesting that they may have started to engraft within the parenchyma, although many remain in the sinusoids. **(A,D)** —× 10 objective, scale = 100 μm; **(B,E)** —× 20 objective, scale = 50 μm; **(C,F)** × 40 objective, scale = 25 μm.

**Figure 6:**
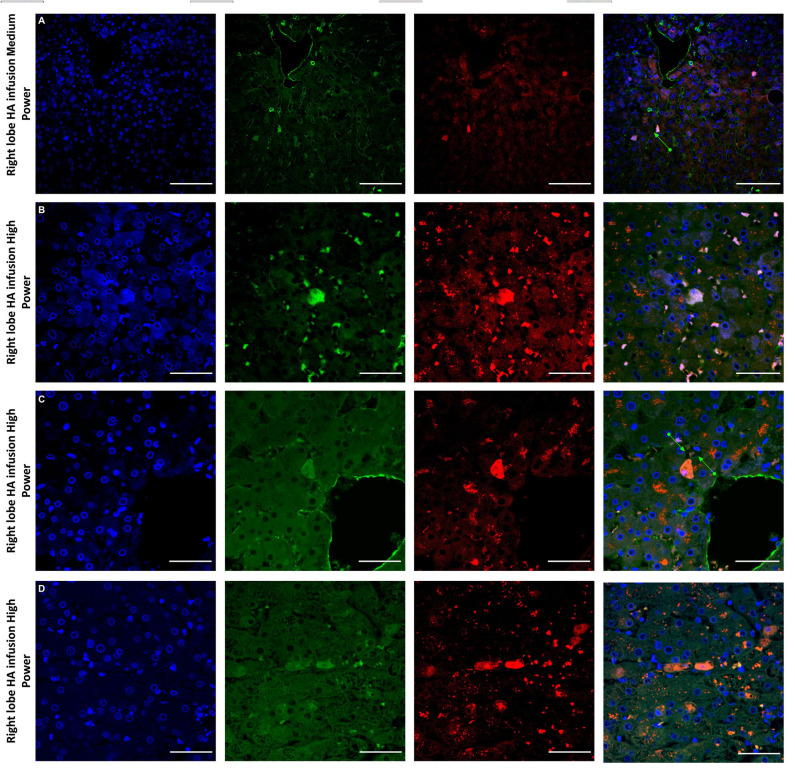
Confocal microscopy images showing representative tissue sections (as labeled) of livers infused with MAPC cells. Blue channel (Nucleic acid probe DAPI 345nm) (4',6-diamidino-2-phenylindole), Red channel (615 nm) CMTPX Red and green channel (FITC 495 nm). Four confocal microscopy panels (blue channel, green channel, red channel, and composite) of representative images of the right lobes of 3 livers infused with MAPC cells via the right hepatic artery after 1 h (**A**—medium power) and 4 h (**B–D**—high power). Here the green arrows in **(A,C)** demonstrate the vascular endothelium stained with CD31 and cells that appear to lie out with the vasculature between the parenchymal cells. These cells also fluoresce in the FITC channel and this may be because they have undergone some form of conformation change during the engraftment process. A × 20 objective, scale = 50 μm; **(B–D)** × 40 objective, scale = 25 μm.

**Figure 7:**
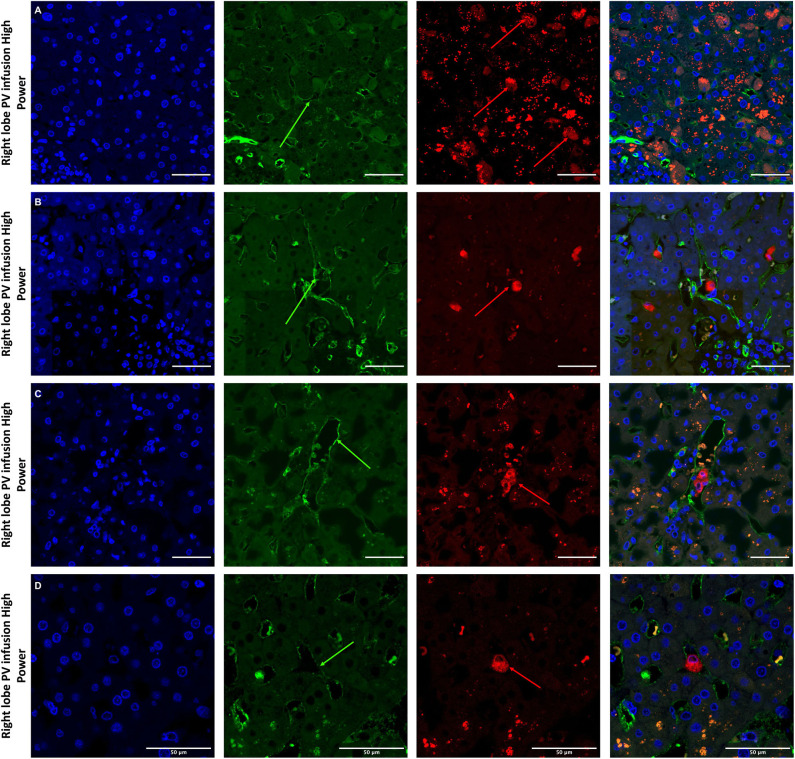
Confocal microscopy images showing representative tissue sections (as labeled) of livers infused with MAPC cells. Blue channel (Nucleic acid probe DAPI 345nm) (4',6-diamidino-2-phenylindole), Red channel (615 nm) CMTPX Red and green channel (FITC 495 nm). Four confocal microscopy panels (blue channel, green channel, red channel, and composite) of representative images of the right lobes of 3 livers infused with MAPC cells via the right portal vein after 1 h (**A**—high power) and 4 h (**B–D**—high power). In this series, the green arrows again demonstrate the vascular endothelium using the CD31 stain but here the MAPC cells are barely visible in the FITC channel and are clearly fluorescing in the red channel suggesting that that are yet to undergo the changes seen in Figure 6. **(A–D)** × 40 objective, scale = 25 μm.

Histological features were in keeping with efficacy of perfusion and liver quality. Architecture of the liver parenchyma was well-maintained in those livers that were deemed viable (Figure 3A). Liver PV3 was severely steatotic and H&E stained sections demonstrated large droplet macrovescicular steatosis and loss of cohesion between hepatocytes in liver cell plates suggesting endothelial disruption (Figure 3B). In those livers that met viability criteria, increases in glycogen storage were observed (Figures 3C,D). Three-color confocal microscopy (4′,6-diamidino-2-phenylindole [DAPI] on the blue channel, CMTPX Red and CD31 on the green channel (to identify vascular endothelium) was used to demonstrate the presence and location of MAPC cells. Cells were visualized in the right lobe of all 6 livers. MAPC cells were visualized in every low power field of view in central and peripheral biopsies of the right lobe (5 random biopsies each of central and peripheral tissue) and were visualized 1 h after infusion and 5 h after infusion [Figures 4, 5, 6, 7 show confocal images comparing right and left lobes pre and post-infusion (4), low power HA vs. PV infusion (5), and high power post HA infusion (6) and post PV infusion (7)]. MAPC cells were never visualized in the left lobe. Arterially infused cells appeared to cross the CD31 stained vascular endothelium and migrate to within the parenchyma. These cells also appear to undergo some form of conformational change as they are also expressed in the green channel in addition to the red channel as opposed to those cells that remain in the vascular channels and are visible in the red channel only.

### Cytokine and Chemokine Analysis of Perfusate Using Luminex

**Figure 8:**
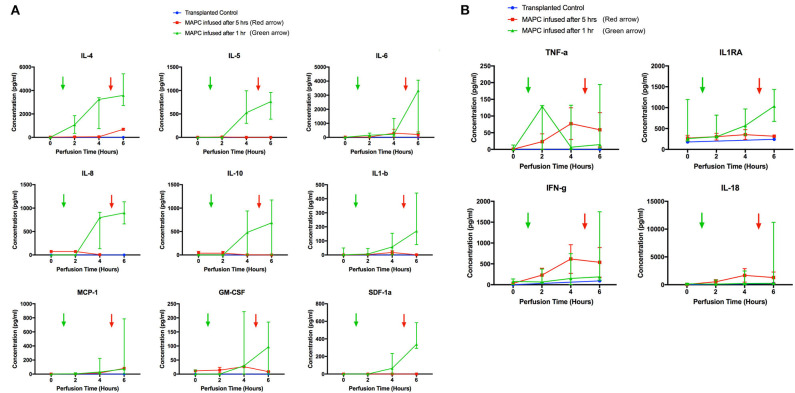
Perfusate analysis using Luminex platform. Pattern of nine targets appear related to cellular perfusion **(A)**, with concentrations increasing in perfusates of livers after the infusion of MAPC cells. Four targets **(B)** appear unrelated to the infusion of MAPC cells. **(A)** shows nine targets that had an apparent increase in concentration within the perfusate samples following MAPC cell administration. IL4 appeared to increase shortly after cell administration—increasing in the final hour of the perfusion after MAPC administration—whereas the remaining targets required longer to increase. As can be seen in the legend the green arrow denotes the administration of MAPC cells at 1 h and the red arrow at 5 h. **(B)** Shows four targets (TFN-alpha, IL1RA, Interferon-gamma, and IL-18) that increased their concentrations during the perfusion and appear unrelated to MAPC cell administration but more likely linked with levels of inflammation within the marginal livers.

From the 34-plex multiplex analysis, the concentrations of 13 out of 34 targets were shown to increase over the course of the perfusion: IL-1RA, IL-1beta, IL4, 5, 6, 8, 10, 18, IFN-gamma, TNF-alpha, MCP-1, GM-CSF, SDF-1 alpha. The results are displayed in Figure 8 (median values with range), with the six livers split into two groups—group 1 (*n* = 2) cells infused after 5 h and group 2 (*n* = 4) cells infused after 1 h. A transplanted control which underwent perfusion was also analyzed at 2 time points (0 and 6 h). The changes in concentration of nine targets (Figure 8A) – IL4, 5, 6, 8, 10, MCP-1, SDF-1 alpha, IL-1 beta, and GM-CSF appeared related to the presence of MAPC cells, as they were only detected after their infusion. The levels of the remaining four targets (Figure 8B) TNF-alpha, IFN-gamma, IL-18, and IL-1RA appeared unrelated to the presence of MAPC cells.

### Proteomic Analysis of Perfusates

**Figure 9:**
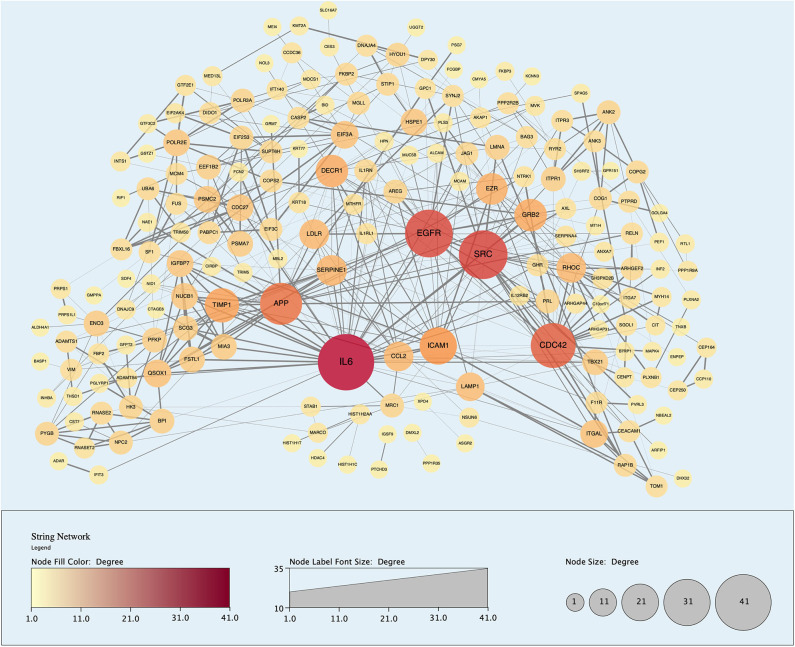
Protein-protein interaction (PPI) network demonstrating 191 unique proteins (nodes) identified in the perfusate of livers infused with MAPC cells during NMP-L. Node size and color is proportional to the number of Interactions associated with said protein. “Edges” or Interactions are based on high confidence of interaction (String database confidence score > 0.700).

**Figure 10:**
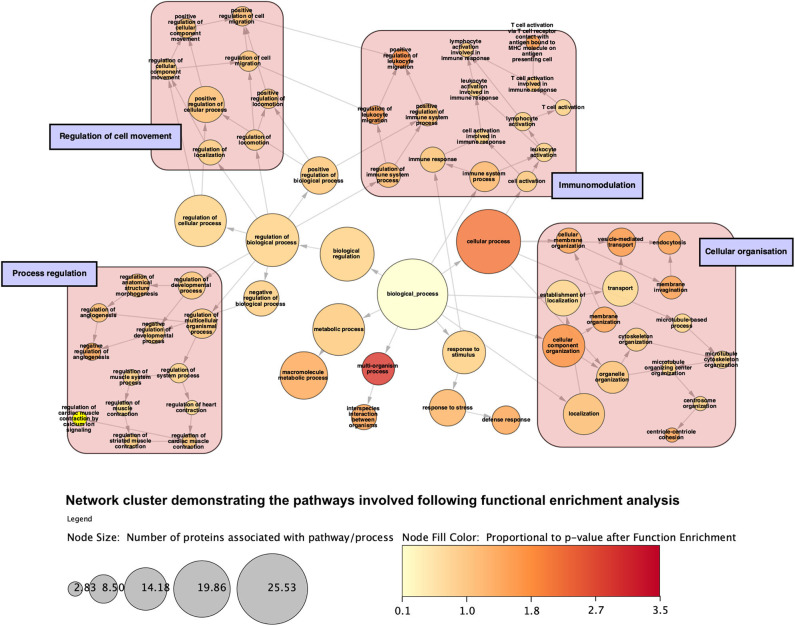
Network cluster demonstrating categories of gene ontology processes that the proteins are involved with following function enrichment analysis. Proteins are grossly involved with regulation of a range of biological a cellular processes, immunomodulation, cellular movement, and compartment organization. Node size is proportional to the number of proteins involved with said process.

**Figure 11:**
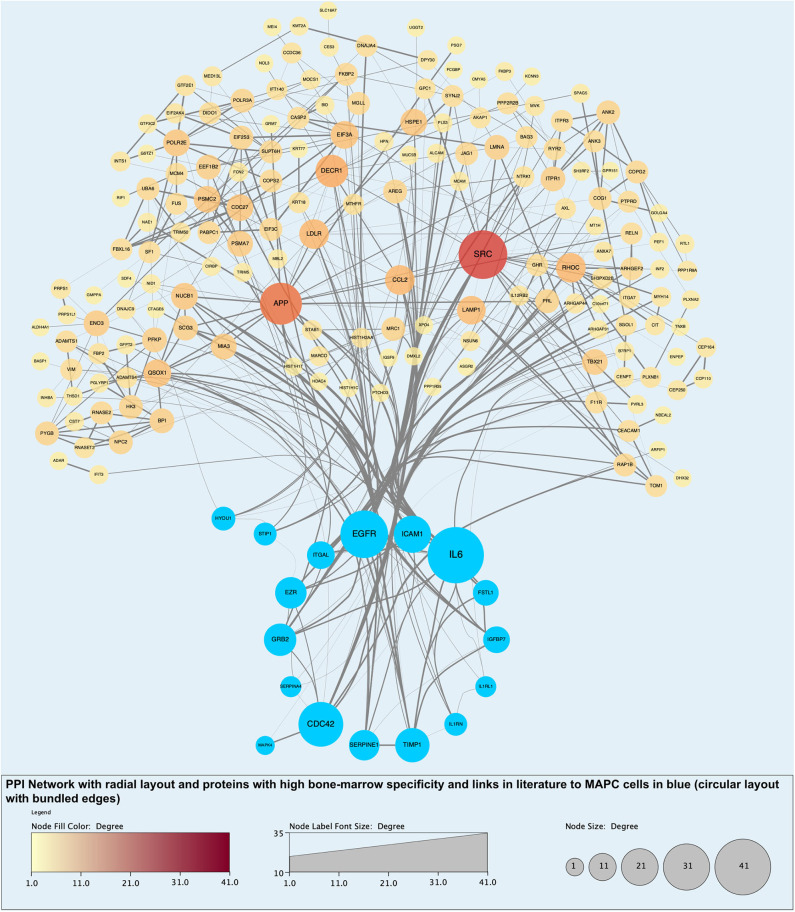
Protein-protein interaction (PPI) network highlighting proteins with evidential links to MAPC cells and MSC in the literature (blue nodes). Of note, these proteins are some of those with the largest number of interactions and roles in biological processes.

Analysis of perfusates from the 6 donor livers identified a total of 1,300 unique proteins of which 48 were present in every sample. Of interest these included alcohol dehydrogenase Ib and 4, superoxide dismutase 1, aldehyde dehydrogenase, complement component 3, apolipoproteins A-II, B and H, glyceraldehyde-3-phosphate dehydrogenase, serpin peptidase inhibitor clade G member 1, kininogen 1 and inter-alpha-trypsin inhibitor heavy chain family, member 4. When the results from these perfusions were compared to a group of 8 contemporaneous perfusions with similar demographics and characteristics that had not received therapeutic intervention, 295 unique proteins were identified in the perfusate from time-points following the infusion of cellular therapy (i.e., after 5 h for HA1 and PV1 and after 1 h in HA2 and 3 and PV2 and 3). The network edges were set to high confidence (>0.700 interaction score) which yielded a PPI enrichment *p*-value of 1.05e−05 showing that it was highly likely that this group of proteins were biologically connected. Unconnected nodes were removed and 191 proteins were imported to Cytoscape for further functional enrichment and network analyses. These proteins (Figure 9), through functional enrichment analysis, were shown to be involved with 549 gene ontology processes (GO:Processes) [false discovery rate [FDR] <0.05]. These are grouped and depicted in Figure 10. Seventeen of these proteins were also identified as having strong links to MAPC cells and MSC in the literature (Figure 11)—with 14 of 17 in the top 50 most connected proteins in terms of “node degree” or PPI (Supplementary Table 1). Many of these had strong tissue associations with the bone marrow and the liver (Supplementary Table 3). The descriptions of these proteins can be found in Table 1 whilst the functional enrichment data can be seen in Supplementary Tables 1–3.

**Table 1:** Descriptions of proteins identified unique to perfusate following MAPC cells administration with links in the literature to MAPC cell and MSC activity.

**Protein**	**Description**
IL6	B-cell stimulatory factor 2; Cytokine with a wide variety of biological functions. It is a potent inducer of the acute phase response.
EGFR	Receptor tyrosine kinase binding ligands of the EGF family and activating several signaling cascades to convert extracellular cues into appropriate cellular responses.
CDC42	Cell division control protein 42 homolog; Plasma membrane-associated small GTPase which cycles between an active GTP-bound and an inactive GDP-bound state.
ICAM1	Intercellular adhesion molecule 1; ICAM proteins are ligands for the leukocyte adhesion protein LFA-1 (integrin alpha-L/beta-2).
TIMP1	Tissue inhibitor of metalloproteinases 1; Metalloproteinase inhibitor that functions by forming one to one complexes with target metalloproteinases.
GRB2	Growth factor receptor-bound protein 2; Adapter protein that provides a critical link between cell surface growth factor receptors and the Ras signaling pathway.
EZR	Cytovillin; Probably involved in connections of major cytoskeletal structures to the plasma membrane.
SERPINE1	Serpin peptidase inhibitor, clade E (nexin, plasminogen activator inhibitor type 1), member 1; Serine protease inhibitor. This inhibitor acts as “'bait” for tissue plasminogen activator, urokinase, protein C and matriptase-3/TMPRSS7.
ITGAL	Leukocyte function-associated molecule 1 alpha chain; Integrin alpha-L/beta-2 is a receptor for ICAM1, ICAM2, ICAM3, and ICAM4.
IGFBP7	Insulin-like growth factor binding protein 7; Binds IGF-I and IGF-II with a relatively low affinity. Stimulates prostacyclin (PGI2) production. Stimulates cell adhesion.
FSTL1	Follistatin-related protein 1; May modulate the action of some growth factors on cell proliferation and differentiation.
HYOU1	Hypoxia up-regulated 1. Has a pivotal role in cytoprotective cellular mechanisms triggered by oxygen deprivation. May play a role as a molecular chaperone and participate in protein folding.
IL1RN	Interleukin-1 receptor antagonist protein; Inhibits the activity of interleukin-1 by binding to receptor IL1R1 and preventing its association with the coreceptor IL1RAP for signaling.
STIP1	Transformation-sensitive protein IEF SSP 3521; Acts as a co-chaperone for HSP90AA1. Mediates the association of the molecular chaperones HSPA8/HSC70 and HSP90.
IL1RL1	Interleukin 1 receptor-like 1; Receptor for interleukin-33 (IL-33). Its stimulation recruits MYD88, IRAK1, IRAK4, and TRAF6, followed by phosphorylation of MAPK3/ERK1 and/or MAPK1/ERK2, MAPK14, and MAPK8.
SERPINA4	Serpin peptidase inhibitor, clade A (alpha-1 antiproteinase, antitrypsin), member 4; Inhibits human amidolytic and kininogenase activities of tissue kallikrein.
MAPK4	Extracellular signal-regulated kinase 4; Atypical MAPK protein. Phosphorylates microtubule- associated protein 2 (MAP2) and MAPKAPK5. May promote entry in the cell cycle.

## Discussion

This is the first study to demonstrate the feasibility and potential advantages of using NMP-L to deliver stem cell therapy to marginal human donor livers. Our data demonstrate that delivery of MAPC cells to human donor livers is feasible, has no detrimental effect on flow or resistance, cells infused via the artery appear to undergo transendothelial migration and there is evidence of beneficial biological activity.

MAPC cells are a distinct bone-marrow derived cellular population that share properties associated with MSC. Unlike standard MSC culture conditions however, they prefer hypoxic conditions in media supplemented with epidermal growth factor and platelet-derived growth factor. MAPC cells have been shown to be non-immunogenic and exert strong immunosuppressive effects on T-cells *in vitro* and may also supress an ongoing immune response (12, 42). These findings paved the way for the use of MAPC cells in models of graft-versus host disease and as an anti-inflammatory therapeutic treatment in models of transplantation. MAPC cells were chosen for this study because they share many of the positive properties of MSC, and a clinical grade version of MAPC cells, MultiStem® cells, have been evaluated in several clinical trials and are easily scalable for use in future NMP-L clinical trials (43–45).

Most animal studies using stem cells in models of liver transplantation deliver the cells either systemically intravenously (where most cells are trapped in the lungs) or via the portal vein—a route that was used for a safety and feasibility study in human subjects (16). Indeed, the portal venous route is also the preferred route for islet cell infusion for the treatment of Type-I diabetes although increased portal venous resistance has been demonstrated (46). The argument for systemic infusion is that the cells appear to exert effects through paracrine mechanisms and soluble mediators (47). Despite this, their effects appear to be strengthened when cell–cell contact is present (42). This points to the presence of cell contact-dependent suppressive activity or suggests that the interaction of immune cells to MAPC cells upregulates their suppressive function through other soluble factors. The process of machine perfusion provides a valuable window of opportunity to deliver cellular therapy directly into the target donor organ, ensuring the presence of the anti-inflammatory therapy before the onset of the immune response during organ reperfusion at clinical transplantation. In this study, there was no evidence of increased resistance or reduced flows when cells were infused via either vascular route. The transient increases in arterial flow are addressed later in the discussion.

Cells were easily identified using fluorescence microscopy, although cells never appeared in the left lobe suggesting that cells became trapped in the disposable circuit if they did not engraft on the first pass. There appeared to be a difference in MAPC cell homing depending on route of infusion with cells infused via the portal vein “arresting” within the sinusoidal channels (localization) whereas arterially-infused cells transmigrated across the vascular endothelium (homing) (Figures 5, 6, 7). These cells also appeared to undergo some form of conformational change possibly through “inside-out” signaling or changes in integrin conformation (48). They fluoresce in the green channel as well as the red after crossing the vascular endothelium to reside within the parenchyma (Figure 7). This observation is similar to that seen in flow assays when migrated cells go from phase light to phase dark and may well influence fluorescent spectral overlap during confocal microscopy.

Hepatic sinusoidal endothelium differs from vascular endothelium in terms of structure and adhesion molecule expression. Despite hepatic sinusoidal endothelium having increased expression of intercellular adhesion molecule-1 (ICAM-1), the absence of cell-cell junctions and reduction in p- and e-selectin expression may reduce the chances of MAPC transmigration across sinusoidal endothelium when infused via the portal route. Cells infused via the artery must pass through a narrow pre- or inter-sinusoidal confluence which may improve their changes of retention within the tissue. The arterial system also supplies the bile ducts and presence of cells near the bile ducts may help ameliorate the bile duct endothelial damage that can occur at reperfusion.

When looking for evidence of MAPC cell functional activity, Luminex analysis of perfusates from different time points yielded some interesting results. Of the 35 intended targets, 13 were detectable in the perfusate. Four of these appeared to be related to graft quality and not the presence of cells although TNF-a, IFN-gamma, and IL1-RA have been shown to upregulate the immunomodulatory effects of stem cells. TNF-a and IFN-gamma, which drive inflammatory and immune mediated responses via activation of macrophages and induction of MHC-II molecules, increased over the course of the perfusion. In combination, they have been shown to increase the immunosuppressive effects of MAPC cells through indoleamine 2,3 dioxygenase activation (49, 50). IL-1RA has also been shown to be an effective anti-inflammatory mediator when used in combination with MSC in models of acute liver failure (51). As mentioned, the concentrations of 9 targets appeared to be related to the timing of MAPC cell infusion. IL4 has been shown to suppresses liver TNF-a mRNA expression, neutrophil accumulation and liver injury (52) whilst IL-10 has been shown to protect against hepatic ischemia-reperfusion injury by suppressing NFkB activation and subsequent expression of pro-inflammatory mediators (53) and importantly both have been shown to be upregulated following MAPC cell administration (54, 55). MCP-1 (CCL2) expression appeared to correlate with cell infusion and has been shown to be secreted by MAPC cells (56). Stimulation of MAPC cells using TNF-a and IFN-g increases expression of chemokine receptor type 2 and promotes migration of the cells to areas of inflammation where MCP-1 (CCL2) is being secreted. This stimulation also increases transcription of iNOS and cyclooxygenase-2 mRNA which leads to production of NO and PGE which are involved mechanistically in the suppression of T-cell proliferation (57, 58). The presence of NO in the MAPC cells-containing media may explain the transient increase in arterial flow and decreased vascular resistance when cells were infused into the right lobe, which subsided within 10 min of the infusion stopping (59). The precise mechanistic relevance of these observations are not clear at present and remain the subject of ongoing research in our group. However, a potential explanation is that the anti-inflammatory response may be liver centric and attempting to reduce the extent of parenchymal injury whilst the increase in inflammatory markers is allowing the potential influx of immune cells that are required for later liver injury resolution (60, 61).

To determine the presence of potentially unique MAPC cell-associated proteins, proteomic analysis of the individual perfusate samples taken after cell infusion was compared to those samples pre-infusion and to eight similar livers that did not receive cellular therapy. The analysis as described in the results section would suggest that MAPC cells, in the presence of a pro-inflammatory environment as confirmed by multiplex analysis, secrete molecules that regulate the biological activity of the extracellular matrix as well as chemokines, cytokines, and molecules that participate in and regulate a variety of biological pathways (Figure 10 and Table 1). Many of these proteins have previously been described in the secretome of MAPC cells and could play an important role in a pro-inflammatory environment, during for example, ischemia-reperfusion (62). The expression of HYOU1 suggests that MAPC cells may be involved in the enhancing the cytoprotective mechanisms within the liver during NMP-L (63). In addition MAPC cells increase the expression of known cell cycle proteins such as GRB2, MAPK4 and the growth factor EGFR. Furthermore, proteins involved in tissue injury resolution such as TIMP-1 and STIP1 are also upregulated suggesting that MAPC cells may regulate this part of the IRI process too (64). The expression of ITGAL and ICAM-1 suggests a potential immuo-modulatory role for MAPC cells although this needs further experimental clarification (65).

We are aware of several limitations in this study, in particular the number of livers included, the timings of the infusions and the different routes of delivery, all of which in combination impact upon the statistical power of the study. We spent a long time considering how best to carry out this research in a cohort of organs that are scarce and generally very heterogenous in nature. Importantly it is precisely such organs that may benefit from this type of therapeutic approach in future. In terms of research, livers obviously differ to kidneys in terms of blood supply and the number in the body. The use of discarded kidneys affords the researcher the opportunity to use one for the intervention and one as a control. Nor is there the need to consider the blood supply to use for delivery of the therapy. In contrast in livers, we must consider the optimal route for delivery and also try to create some form of internal control as discarded human livers are too heterogenous to be able to draw robust statistical conclusions given the limited numbers offered for scientific research. We were also unable to comment on the effect of MAPC cell delivery on overall organ “viability” or the ability of the MAPC cells to “rescue” an organ currently deemed untransplantable. In this regard, multiple factors are at play in terms of overall organ viability. It is likely that the mechanisms at play may not significantly impact upon gross organ viability but are more likely to attenuate the inflammatory and immune responses at a cellular level and this would hopefully translate into improved outcomes following *in-situ* reperfusion.

This research, as stated in the aims was a pilot study that set out to (a) develop a technique for infusion and demonstrate the feasibility of NMP-L to deliver cellular therapy to extended criteria human donor livers; (b) determine the best vascular route for delivery and confirm the presence of cellular engraftment and (c) determine parameters that may reflect biologically functional activity imparted by the presence of the therapeutically administered MAPC cells. Whilst we recognize that the comparatively small n-numbers and differences of timing of infusion of the cells were potential limitations to our study, we nevertheless believe that the techniques and the data obtained are sufficiently robust to permit cautious but valid analysis and conclusions.

## Conclusion

This is the first study to investigate the feasibility of using machine perfusion to deliver cellular therapy to human donor livers. We have demonstrated that cells can be delivered directly to the target organ without compromising the perfusion. This not only overcomes the disadvantages associated with systemic infusion, but ensures the cells are present before ingress of the recipient immune cell population. The arterial route of infusion appears to result in more effective cellular engraftment. MAPC cells secrete a host of soluble factors that are known to have anti-inflammatory and immunomodulatory effects that would be especially beneficial for extended criteria donor livers.

## Data Availability Statement

The raw data supporting the conclusions of this article will be made available by the authors, without undue reservation, to any qualified researcher.

## Ethics Statement

Ethical approval for the study was granted by the National Research Ethics Service committee in London-Surrey Borders (reference number 13/LO/1928). Consent for the use of donor tissues for research was obtained by the specialist nurses in organ donation from the designated donor's next of kin.

## Author Contributions

RL: conception and design, collection and/or assembly of data, data analysis and interpretation, manuscript writing, final approval of manuscript. SS: conception and design, administrative support, collection and/or assembly of data, data analysis, and interpretation, manuscript writing. LW: collection and/or assembly of data, data analysis, and interpretation. VR: provision of study material or patients, data analysis, and interpretation, manuscript writing. RB: data analysis and interpretation, manuscript writing. AS: data analysis and interpretation, manuscript writing. YB: collection and/or assembly of data. GR: collection and/or assembly of data, data analysis, and interpretation. AT: conception and design, manuscript writing, final approval of manuscript. DM: conception and design, manuscript writing. PN: conception and design, manuscript writing. HM: collection and/or assembly of data, provision of study material or patients. SA: conception and design, manuscript writing, final approval of manuscript. All authors contributed to the article and approved the submitted version.

## Conflict of Interest

AT is an employee of Athersys Inc. SS was an employee of Athersys at the time the work was completed. VR is employed by ReGenesys BVBA. The remaining authors declare that the research was conducted in the absence of any commercial or financial relationships that could be construed as a potential conflict of interest.

## Abbreviations

CD: cluster of differentiation
DBD: donation following brain death
DCD: donation following circulatory death
HA: hepatic artery
ICAM-1: intercellular adhesion molecule-1
IRI: ischaemia reperfusion injury
MAPC cells: multipotent adult progenitor cells
MSC: mesenchymal stem cells
NHS: National Health Service
NHSBT: National Health Service Blood and Transplant
NMP-L: normothermic machine perfusion of the liver
PV: portal vein.
