# Supplementary material for: The Delivery of Multipotent Adult Progenitor Cells to Extended Criteria Human Donor Livers Using Normothermic Machine Perfusion
Source: Front Immunol. 2020 Jun 25;11:1226. doi: 10.3389/fimmu.2020.01226 (PMC7344318; doi:10.3389/fimmu.2020.01226)
Supplement: Supplementary file 1 [file Table_1.DOCX]

| **Supplementary Table 1.** Analysis of 17 proteins with links to MAPC cells and MSC in the literature using Cytoscape. | | | | | | | |
| --- | --- | --- | --- | --- | --- | --- | --- |
| **Protein** | **Interaction Rank** | **Betweenness Centrality** | **Closeness Centrality** | **Clustering Coefficient** | **Degree** | **Neighbourhood Connectivity** | **Accession Number** |
| IL6 | 1 | 0.241 | 0.463 | 0.148 | 41 | 10.39 | P05231 |
| EGFR | 3 | 0.189 | 0.458 | 0.165 | 32 | 11.66 | Q9H2C9 |
| CDC42 | 4 | 0.163 | 0.427 | 0.128 | 29 | 9.69 | P60953 |
| ICAM1 | 6 | 0.051 | 0.409 | 0.252 | 21 | 13.38 | P05362 |
| TIMP1 | 7 | 0.023 | 0.376 | 0.373 | 18 | 13.44 | P01033 |
| GRB2 | 8 | 0.054 | 0.412 | 0.258 | 16 | 14.44 | P62993 |
| EZR | 10 | 0.064 | 0.390 | 0.219 | 15 | 13.27 | P15311 |
| SERPINE1 | 11 | 0.016 | 0.384 | 0.473 | 14 | 17.43 | P05121 |
| ITGAL | 16 | 0.009 | 0.349 | 0.382 | 11 | 14.00 | P20701 |
| IGFBP7 | 19 | 0.007 | 0.338 | 0.711 | 10 | 14.80 | Q16270 |
| FSTL1 | 25 | 0.002 | 0.336 | 0.806 | 9 | 15.33 | Q12841 |
| HYOU1 | 36 | 0.016 | 0.292 | 0.190 | 7 | 5.14 | Q9Y4L1 |
| IL1RN | 46 | 0.002 | 0.330 | 0.467 | 6 | 16.67 | P18510 |
| STIP1 | 47 | 0.007 | 0.326 | 0.400 | 6 | 9.00 | P31948 |
| IL1RL1 | 82 | 9.44E-05 | 0.320 | 0.500 | 4 | 15.75 | Q01638 |
| SERPINA4 | 83 | 6.31E-05 | 0.336 | 0.833 | 4 | 24.75 | P29622 |
| MAPK4 | 122 | 4.23E-04 | 0.303 | 0.000 | 2 | 17.00 | P31152 |
