# Supplementary material for: The Delivery of Multipotent Adult Progenitor Cells to Extended Criteria Human Donor Livers Using Normothermic Machine Perfusion
Source: Front Immunol. 2020 Jun 25;11:1226. doi: 10.3389/fimmu.2020.01226 (PMC7344318; doi:10.3389/fimmu.2020.01226)
Supplement: Supplementary file 2 [file Table_2.DOCX]

| **Supplementary Table 2.** Analysis association with tissue types of 17 proteins identified, with links to MAPC cells and MSC in the literature using Cytoscape. The analysis shows the high affinity of some of the proteins for liver and bone marrow tissue. | | | | | | | |
| --- | --- | --- | --- | --- | --- | --- | --- |
|  | **Tissue Association** | | | | | | |
| **Protein** | **Blood** | **Bone marrow** | **Intestine** | **Kidney** | **Liver** | **Lung** | **Spleen** |
| IL6 | 3.918653 | 3.707035 | 3.74526 | 3.165083 | 3.650198 | 4.847484 | 3.552847 |
| EGFR | 3.107672 | 2.458089 | 3.563364 | 3.261474 | 4.720948 | 3.374401 | 2.973813 |
| CDC42 | 4.863896 | 3.196989 | 3.647546 | 4.279603 | 3.263513 | 4.30881 | 3.330003 |
| ICAM1 | 4.737942 | 4.679953 | 3.26731 | 4.60969 | 4.691104 | 4.959892 | 3.788965 |
| TIMP1 | 4.692988 | 4.617691 | 3.790703 | 3.386743 | 3.687787 | 4.853358 | 3.464487 |
| GRB2 | 4.657075 | 3.150647 | 3.148853 | 3.156573 | 3.319363 | 4.865175 | 3.031142 |
| EZR | 3.024185 | 2.792432 | 4.969846 | 4.81621 | 3.44159 | 4.420051 | 3.301009 |
| SERPINE1 | 3.547812 | 2.039812 | 3.167064 | 3.13832 | 4.324948 | 4.830076 | 2.521216 |
| ITGAL | 3.40197 | 3.010623 | 2.826971 | 2.152066 | 2.310327 | 2.582356 | 3.337133 |
| IGFBP7 | 2.347871 | 2.696723 | 3.946526 | 3.646674 | 3.184157 | 3.302853 | 3.127803 |
| FSTL1 | 2.419277 | 4.358165 | 2.774837 | 4.775193 | 2.714848 | 3.660654 | 3.017779 |
| HYOU1 | 4.515013 | 4.423618 | 3.726329 | 4.020683 | 4.803292 | 3.948119 | 2.567119 |
| IL1RN | 3.484812 | 4.664049 | 2.953125 | 2.249941 | 3.062847 | 2.741958 | 2.559191 |
| STIP1 | 3.115767 | 2.036527 | 4.161274 | 2.649974 | 4.665295 | 4.709531 | 2.188149 |
| IL1RL1 | 4.463048 | 4.381139 | 2.319659 | 2.361319 | 2.240113 | 3.18001 | 2.439147 |
| SERPINA4 | 2.459454 | 4.224 | 4.442 | 2.701973 | 3.569866 | 1.841511 | 1.329396 |
| MAPK4 | 1.733858 | 1.412989 | 2.402393 | 2.309261 | 2.427458 | 2.231179 | 2.146079 |
