# Supplementary material for: The Delivery of Multipotent Adult Progenitor Cells to Extended Criteria Human Donor Livers Using Normothermic Machine Perfusion
Source: Front Immunol. 2020 Jun 25;11:1226. doi: 10.3389/fimmu.2020.01226 (PMC7344318; doi:10.3389/fimmu.2020.01226)
Supplement: Supplementary file 3 [file Table_3.DOCX]

| **Supplementary Table 3.** Analysis association with cellular compartment of 17 proteins identified, with links to MAPC cells and MSC in the literature using Cytoscape. | | | | | | | | | | | |
| --- | --- | --- | --- | --- | --- | --- | --- | --- | --- | --- | --- |
|  | **Compartment association** | | | | | | | | | | |
| **Protein** | **Cytoskeleton** | **Cytosol** | **Endoplasmic reticulum** | **Endosome** | **Extracellular** | **Golgi apparatus** | **Lysosome** | **Mitochondrion** | **Nucleus** | **Peroxisome** | **Plasma membrane** |
| **IL6** | 3.354946 | 3.659442 | 4.611653 | 2.931142 | 5 | 2.31369 | 3.06277 | 3.428203 | 3.932258 | 2.669886 | 4.722855 |
| **EGFR** | 3.35739 | 4.117444 | 4.096257 | 5 | 5 | 4.025284 | 3.138361 | 2.935897 | 5 | 2.122962 | 5 |
| **CDC42** | 5 | 4.764543 | 4.466275 | 2.728715 | 4.591196 | 4.004077 | 2.395977 | 2.609172 | 3.439988 | 1.37444 | 5 |
| **ICAM1** | 2.988354 | 2.938765 | 2.407166 | 2.411702 | 5 | 1.848586 | 2.474571 | 2.701092 | 3.286699 | 2.017282 | 4.892737 |
| **TIMP1** | 2.748153 | 2.429379 | 4.423712 | 1.699162 | 5 | 2.717778 | 2.041388 | 2.415094 | 2.844126 | 1.758408 | 2.783159 |
| **GRB2** | 2.719478 | 4.800379 | 2.43886 | 5 | 4.542171 | 3.747823 | 2.221659 | 2.163797 | 5 | 1.251704 | 4.613605 |
| **EZR** | 5 | 5 | 1.974761 | 5 | 4.516862 | 1.89956 | 1.982185 | 1.962349 | 4.70477 | 1.40228 | 5 |
| **SERPINE1** | 2.655127 | 3.471297 | 2.129191 | 1.643996 | 5 | 1.446422 | 2.200859 | 2.444159 | 2.884933 | 2.087754 | 4.565001 |
| **ITGAL** | 2.222182 | 1.935194 | 1.875613 | 1.617751 | 4.526557 | 1.386448 | 1.841888 | 1.805171 | 2.130471 | 0.28125 | 5 |
| **IGFBP7** | 2.014915 | 1.870236 | 4.284489 | 1.383464 | 5 | 2.277696 | 1.552975 | 1.896298 | 2.189255 | 0.956855 | 2.650884 |
| **FSTL1** | 1.788215 | 2.859218 | 4.286644 | 1.660466 | 4.780764 | 2.022792 | 1.36825 | 1.478438 | 1.95462 |  | 1.908227 |
| **HYOU1** | 1.595914 | 2.019739 | 5 | 1.533618 | 4.47633 | 2.341553 | 1.647339 | 1.984172 | 1.819646 | 1.1072 | 1.641448 |
| **IL1RN** | 2.052677 | 3.682477 | 1.811694 | 1.611266 | 5 | 1.027892 | 1.939878 | 2.092753 | 3.033866 | 1.302627 | 4.537062 |
| **STIP1** | 1.898959 | 4.746566 | 1.990733 | 1.244352 | 1.685461 | 4.243705 | 1.364771 | 1.889113 | 5 | 1.180357 | 2.756355 |
| **IL1RL1** | 1.652793 | 4.36579 | 1.272179 | 1.233713 | 5 | 0.951325 | 1.187238 | 1.364088 | 2.263667 | 1.421838 | 5 |
| **SERPINA4** | 2.385003 | 2.441871 | 2.953946 | 2.557145 | 5 | 2.741868 | 2.128396 | 1.991044 | 2.233325 | 1.485331 | 3.128373 |
| **MAPK4** | 1.549089 | 4.542494 | 1.047963 | 0.937485 | 1.485496 | 0.890861 | 0.651552 | 1.479566 | 4.51324 | 0.853315 | 1.455112 |
