# Supplementary material for: The Delivery of Multipotent Adult Progenitor Cells to Extended Criteria Human Donor Livers Using Normothermic Machine Perfusion
Source: Front Immunol. 2020 Jun 25;11:1226. doi: 10.3389/fimmu.2020.01226 (PMC7344318; doi:10.3389/fimmu.2020.01226)

# PURPOSE

## To fluorescently label MAPC cells that are healthy and stable post cryostorage

# Scope and applicability

## Generation of fluorescently labeled banks of MAPC

# Responsibility AND Personnel qualifications

## This procedure was performed by trained scientific staff at Athersys

# Definitions

## MAPC- multi-potent adult progenitor cell

## PPE- Personal Protective Equipment

# Reference Documents

## Cell Track CMTPX Red Fluorescent Probes manual

# Equipment, REAGENTS, AND CONSUMABLES

| 2-20 μL pipette and tips | Serological pipettes | CMPTX Red Cell Tracker (C24552) |
| --- | --- | --- |
| 20-200 μL pipette and tips | Kim wipes | Isotonic solution |
| 100-1000 μL pipette and tips | 70% Ethanol | Fibronectin coated tissue culture flasks/plates |
| Pipetteman | Nitrile gloves | Sterile DMSO |
| Vacuum pump | Bio Safety Cabinet |  |

# Procedure

## Work in a biosafety hood at all times

## Thaw cryopreserved MAPC

### Remove up to 3 vials at a time of MAPC from liquid nitrogen vapor phase

### Tighten tube cap by hand

### Holding tube by top of cap, thaw vial in 37+/- 2◦C water bath with gentle swirling

### Examine tube every 30 seconds for visible evidence of thawing.

### Once no frozen material within tube is visible by eye, immediately remove the vial from the water bath, dry with a Kim Wipe, and move into the hood.

### Spray vial with 70% ethanol and wipe dry with fresh Kim wipe

### Pour contents of cryovial into a 15 ml or 50 ml conical

### Gently and slowly dilute cells with pre-warmed 10 ml of MAPC media (proprietary), starting with a drop at a time and slowly increasing the volume delivered per second, while swirling the conical.

#### If thawing more than one vial, be sure to dilute each set of cells at the same time, delivering about 1ml to each conical, while swirling, and then moving to the next conical, to ensure that all cells are treated the same

### Once the cells have been diluted at least 10 fold with warmed MAPC media, centrifuge at 400x g at room temperature for 5 minutes.

### Resuspend cells in 2 ml MAPC media (proprietary)

## Count cells on a hemocytometer using Trypan Blue

### Record viability and cell count

## Resuspend cells in MAPC media at a concentration of 12650 cells/ml and plate on fibronectin coated tissue culture plates at 2000/cm^2^

### Culture at 37◦C for 2-3 days. No need to change the media during those days.

## Remove cells from plate using trypsin digestion (5 min at 37◦C with tapping side of flask halfway through)

### Transfer cells to a 50 ml conical and dilute to 30 ml with MAPC media.

### Centrifuge cells at 400xg for 5 min at room temp.

## Cell Tracker CMTPX label cells as detailed in manufacturer’s protocol

### Pour of supernatant and resuspend in prewarmed serum free media (PBS, saline, DMEM are all fine) at 1 million cells/ml

### Bring CMTPX and DMSO to room temp

### Add DMSO to CMPTPX Cell Tracker to final concentration of 10mM

### Use at 10µM (1:1000 dilution)

### Incubate Cell Tracker CMTPX with cells for 30 at culture conditions (37◦C)

### Add 2ml media and centrifuge at 400x g for 5 min at room temp

### Discard supernatant and add 2ml media and centrifuge at 400x g for 5 min at room temp

### Discard Supernantant and Resuspend in freezing media (MAPC media supplemented with 5% DMSO)

### Reserve 50,000 cells for flow cytometric analysis

#### Add 100ul PBS and 1ul 7-AAD to sample and analyze % viability and % labeled via flow cytometry

### Freeze down at 2-10 million cells per ml

### 7.5.10.1 Use control temperature freezers, place into -80C for 1 day and then transfer to liquid nitrogen

# QA/QC

## Cells were analyzed by microscopy and flow cytometery after staining and after freezing/thawing for viability, morphology, and percent labeled.

# INterferences

## DMSO is toxic to cells. Keep cells on ice when in contact with DMSO

# Cautions

## Always treat human cells and cell lines using Universal Precautions

# Health and safety warnings

## Always wash your hands before and after removing gloves when working with blood. Make sure any open wounds are covered with an adhesive bandage.

## If working with any sharps avoid recapping and dispose of in sharps container

## Decontaminate all equipment and devices that come into contact with human cells with 10% bleach solution

## Refer to MSDS sheets for any reagents, as needed

# Associated Documents

## See Appendix A

# Results

## Flow cytometric results of Viability and Dye Retention – Table 1 – Of freshly stained and unstained cells and then of stained cells after 3 and 14 days of cryopreservation.

|  | Fresh Unstained | Fresh Stained | Frozen Stained 3d | Frozen Stained 14d |
| --- | --- | --- | --- | --- |
|  |  |  |  |  |
| % Viable | 99.6 | 94.1 | 98.0 | 95.1 |
| % CMPTX + | 0 | 97.7 | 95.5 | 95.0 |

## Example raw data from FACSCantoII machine [live/dead staining (P2 is viable gate) and CMPTX Red (P3 is stained gate)]

##
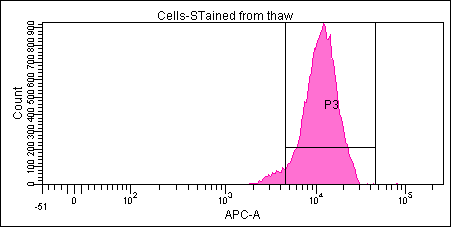

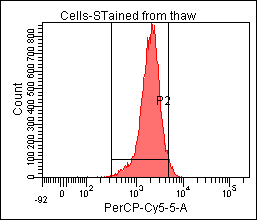

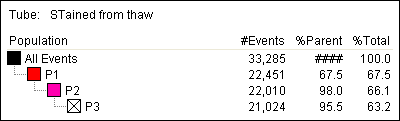


APPENDIX A


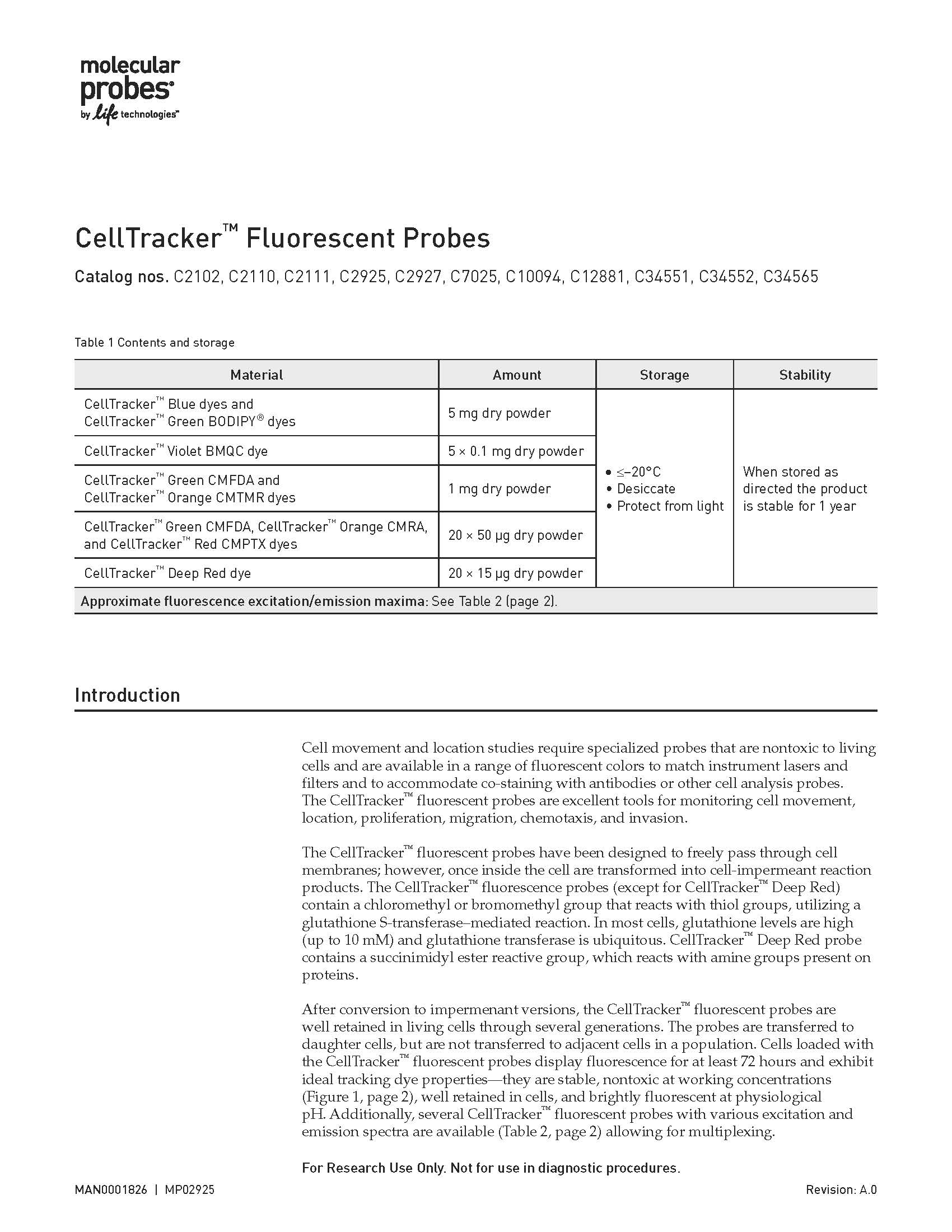

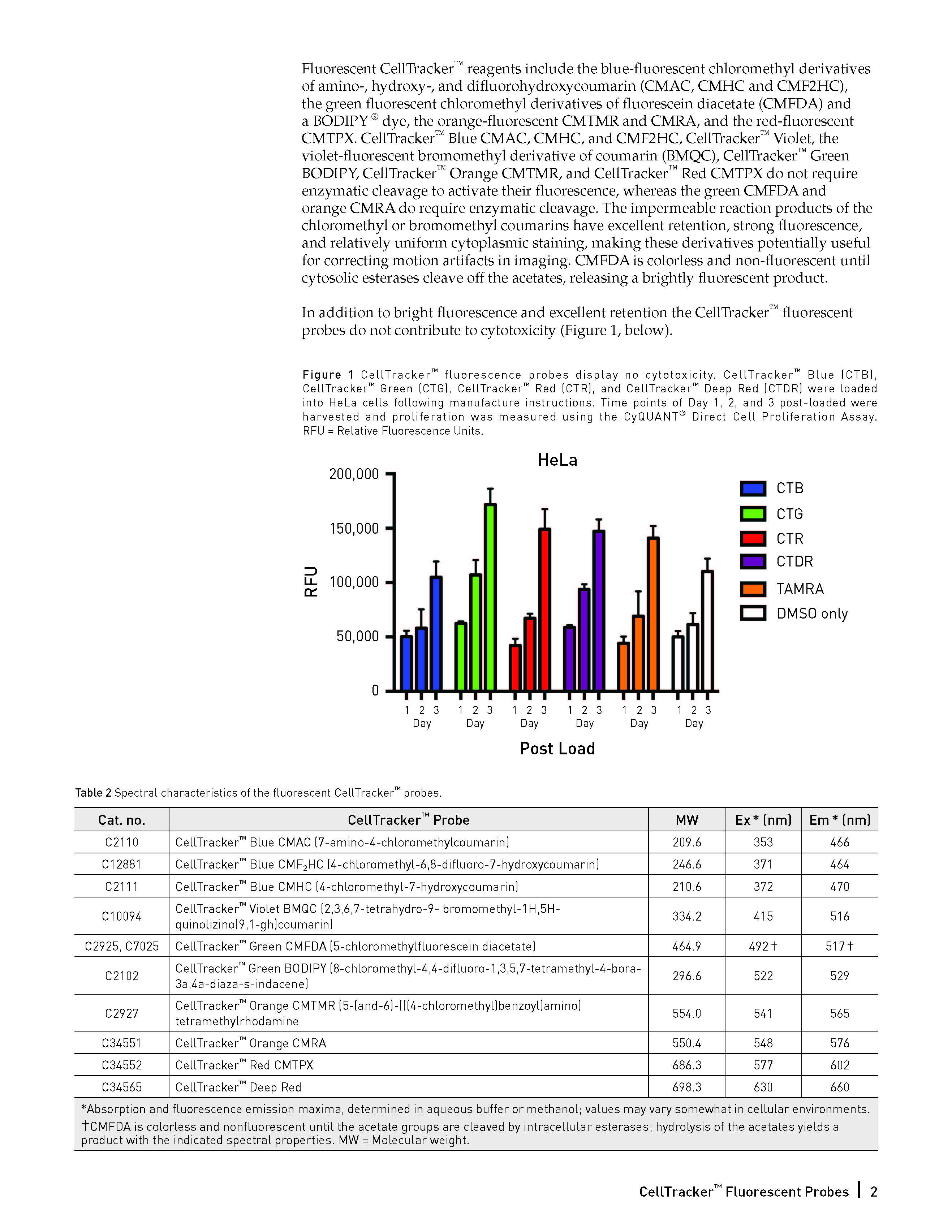

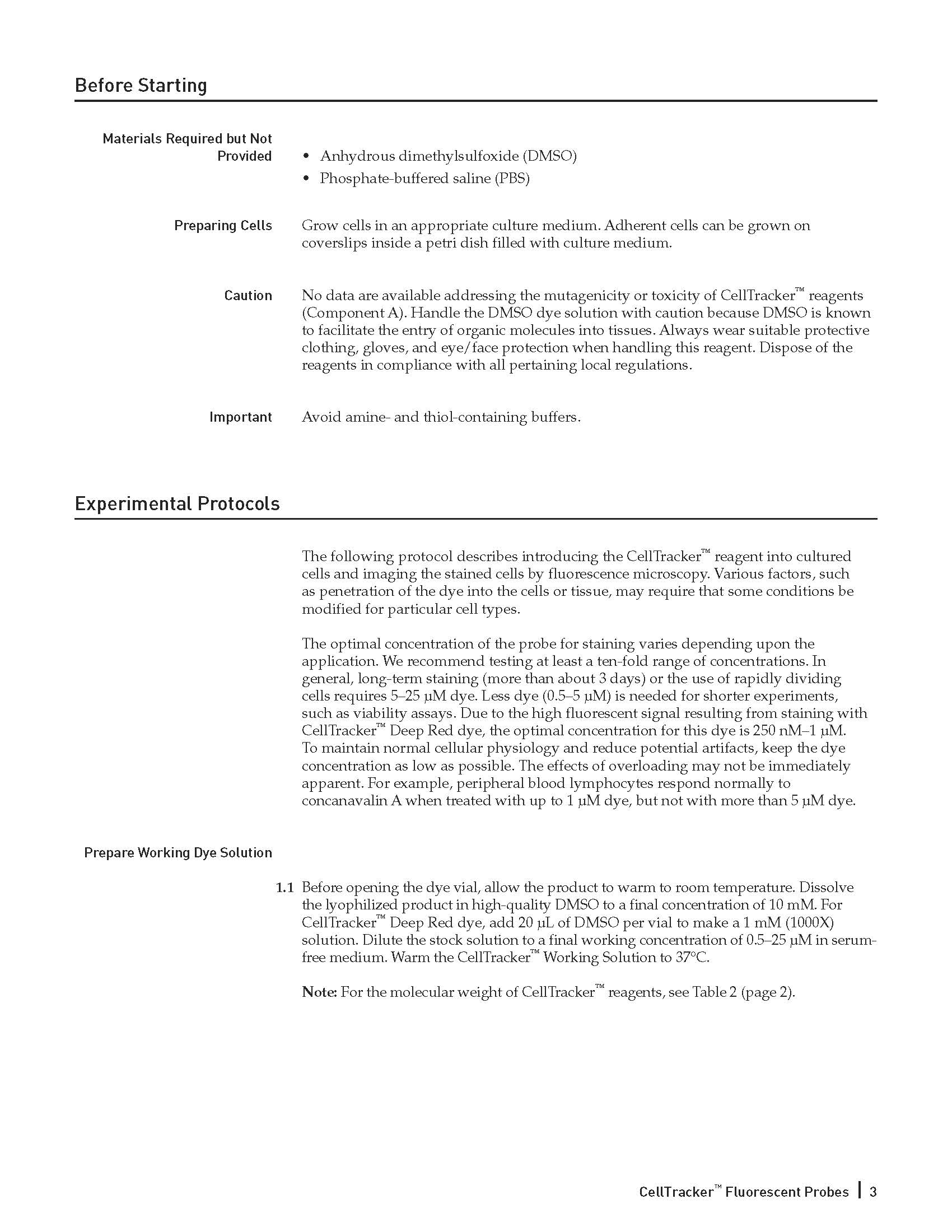

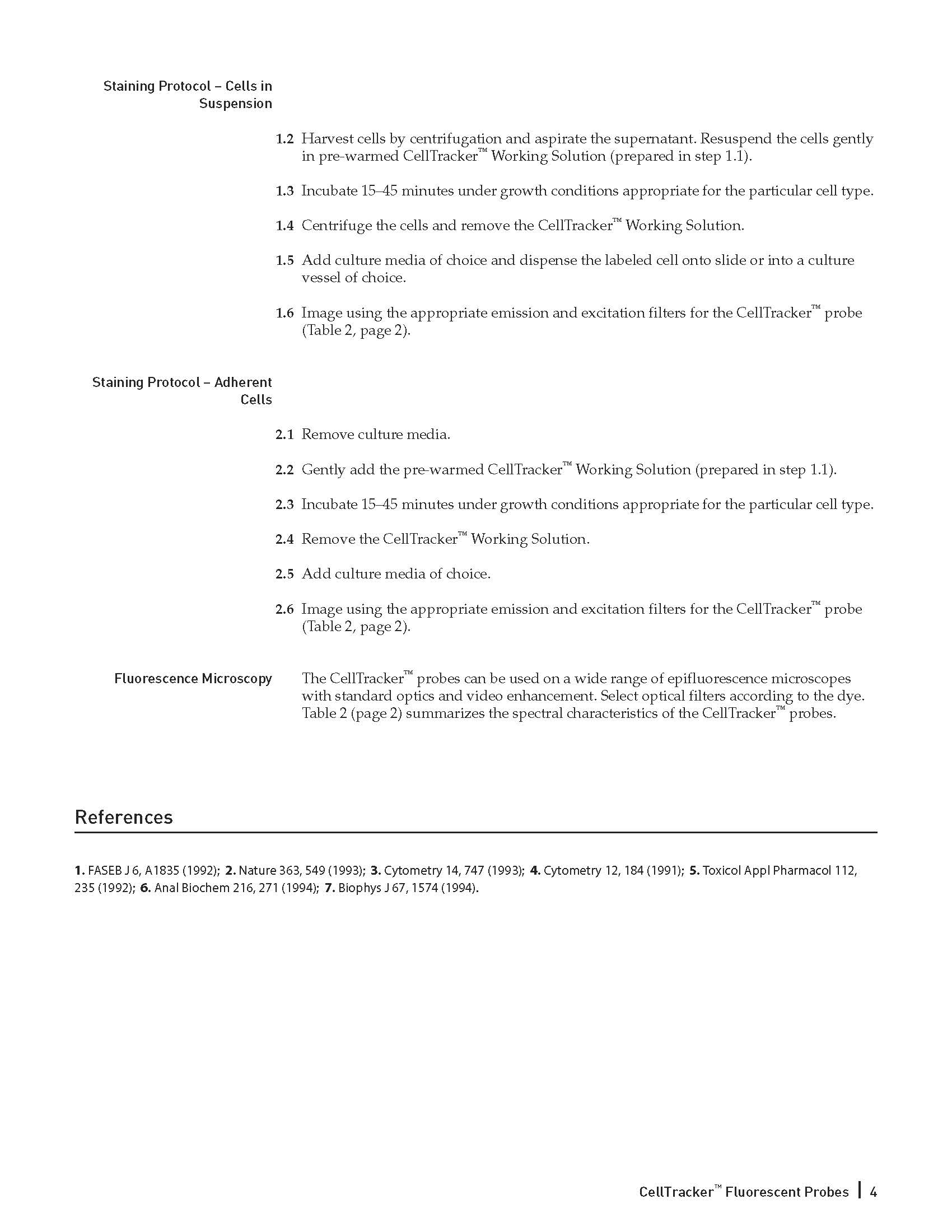

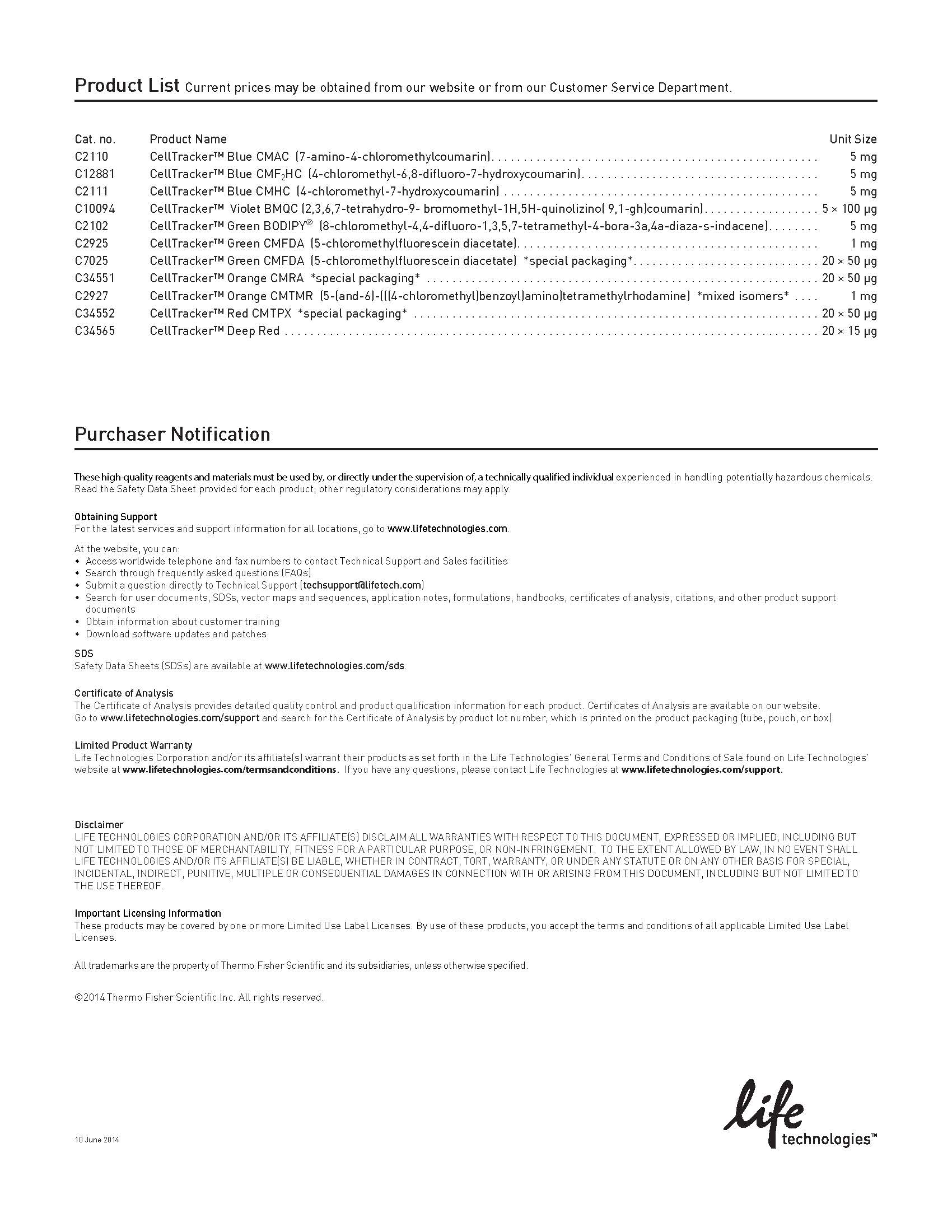

Supplement: Supplementary file 4 [file Data_Sheet_1.docx]
