# Supplementary material for: The Delivery of Multipotent Adult Progenitor Cells to Extended Criteria Human Donor Livers Using Normothermic Machine Perfusion
Source: Front Immunol. 2020 Jun 25;11:1226. doi: 10.3389/fimmu.2020.01226 (PMC7344318; doi:10.3389/fimmu.2020.01226)
Supplement: Supplementary file 5 [file Data_Sheet_2.docx]

Date: 17 Sept 2014

From: Samantha Stubblefield, Ph.D.

To: Richard Laing

Re: Procedure for thawing and diluting MultiStem® into mice for nonclinical research studies

**1 EQUIPMENT, REAGENTS AND CONSUMABLES**

| 1000 μL pipette and sterile filtered tips |
| --- |
| 1 ml 27g insulin syringes (Fisher # 22-272-382 or equivalent) |
| 3ml syringes with 16g needle |
| Ice bucket |
| Ice |
| Water bath with water heated to 37±2°C |
| Kim wipes |
| PBS/Saline/Isotonic Solution |
| 70% Ethanol |
| 50 ml conical |
| P1000 hand pipette |

**2 PROCEDURE**

2.1 Store cryopreserved MultiStem

2.1.1 Store cryopreserved MultiStem in liquid nitrogen vapor phase.

2.2 Thaw cryopreserved MultiStem

2.2.1 Determine number of vials to be thawed

2.2.1.1 Once thawed, vials of MultiStem can be stored on ice for up to 2 hours prior to use

2.2.1.2 Thawed vials of MultiStem cannot be used for animal infusions after 2 hours on ice

2.2.2 Remove up to 3 vials of MultiStem from liquid nitrogen vapor phase

2.2.3 Tighten tube cap by hand

2.2.4 Holding tube by top of cap, thaw vial in 37±2°C water bath with gentle swirling

2.2.5 Examine tube by eye every 30 seconds for visible evidence of thawing

2.2.6 Once no frozen material within tube is visible by eye, immediately remove vial from water bath and dry tube with Kim wipes

2.2.7 Ensure tightness of tube cap by hand

- - 1. Spray tube with 70% ethanol and wipe dry with fresh Kim wipes
    2. Record time of day MultiStem was thawed.
  1. Dilution of MultiStem
     1. Using a 3 ml syringe and 16 g needle or P1000 pipette, draw up the contents of the vial into the syringe and transfer to 50ml conical tube
     2. Using a 3 ml syringe and 16 g needle or P1000, rinse the vial using 1 ml of vehicle formulation per vial.
     3. Combine the rinse with the cells in a 50 ml conical tube.
     4. Determine the cell concentration and viability manually by trypan blue exclusion, being sure to only count viable (non-blue) cells.
     5. Record cell count and viability. Note the time of day at which vial was thawed
     6. Then dilute cells to 10 x 10^6^ cells in 20 ml total of saline, PBS, isotonic solution
        1. Thawed vials of MultiStem cannot be used for animal infusions after 4 hours on ice

**3 HEALTH AND SAFETY WARNINGS**

3.1 Always wear gloves and lab coat while working with cell samples.

3.2 No hazardous chemicals are used in this assay.

3.3 Take basic laboratory safety precautions and wear all PPE.

3.4 Refer to MSDS sheets of any reagents used as necessary.
